# Project Gatekeeper: An Entrance Control System Embedded Radiation Detection Capability for Security Applications

**DOI:** 10.3390/s20102957

**Published:** 2020-05-23

**Authors:** Peter G. Martin, Yannick Verbelen, Elia Sciama Bandel, Mark Andrews, Thomas B. Scott

**Affiliations:** 1 Interface Analysis Centre, School of Physics, HH Wills Physics Laboratory, University of Bristol, Tyndall Avenue, Bristol BS8 1TL, UK; yannick.verbelen@bristol.ac.uk (Y.V.); t.b.scott@bristol.ac.uk (T.B.S.); 2 School of Physics, HH Wills Physics Laboratory, University of Bristol, Tyndall Avenue, Bristol BS8 1TL, UK; wu18396@bristol.ac.uk; 3 Gunnebo UK Ltd., Fairfax House, Pendeford Business Park, Wolverhampton WV9 5HA, UK; Mark.Andrews@Gunnebo.com

**Keywords:** radiation, nuclear threat reduction, security, portal, Geiger–Muller, gateway, gamma-ray, trafficking

## Abstract

Threat assessments continue to conclude that terrorist groups and individuals as well as those wanting to cause harm to society have the ambition and increasing means to acquire unconventional weapons such as improvised nuclear explosive devices and radiological disposal devices. Such assessments are given credence by public statements of intent by such groups/persons, by reports of attempts to acquire radioactive material and by law enforcement actions which have interdicted, apprehended or prevented attempts to acquire such material. As a mechanism through which to identify radioactive materials being transported on an individual’s person, this work sought to develop a detection system that is of lower-cost, reduced form-factor and more covert than existing infrastructure, while maintaining adequate sensitivity and being retrofittable into an industry standard and widely utilised Gunnebo Speed Gate system. The system developed comprised an array of six off-set Geiger–Muller detectors positioned around the gate, alongside a single scintillator detector for spectroscopy, triggered by the systems inbuilt existing IR proximity sensor. This configuration served to not only reduce the cost for such a system but also allowed for source localisation and identification to be performed. Utilising the current setup, it was possible to detect a 1 µSv/h source carried into the Speed Gate in all test scenarios, alongside locating and spectrally analysing the material in a significant number.

## 1. Introduction

As evidenced by the Doomsday Clock, a “measure of the likelihood of a man-made global catastrophe” being set to just 100-s to midnight—a status never before realised—the world is now more insecure, and humanity’s existence more threatened than ever before [1,2]. Consequently, threat assessments continue to conclude that terrorist groups and state-sponsored actors have the ambition, resource and the driver to acquire, divert or produce materials (whether radiological, nuclear, chemical, biological or explosive) to damage society as part of their malicious intentions [3]. These assessments are given credence by public statements of intent by groups and nations, by reports of and actual attempts to acquire/produce such materials and by law enforcement actions which have interdicted or prevented attempts to acquire such material. Radioactive and nuclear is one category within this “global hazard” and is significant over the others, owing to its substantial inventory within the global nuclear fuel cycle alongside the large amount of naturally occurring radioactive material and specialist radioactive sources. Hence, attempted malicious acts involving the removal of radioactive material from hospitals, universities and nuclear facilities are not unknown and allegations that various terrorist/non-state groups have tried to acquire radioactive materials have been widely reported [1,4,5].

Occurrences of the “illicit trafficking and other unauthorised activities and events involving nuclear and other radioactive material outside of regulatory control” are recorded by the International Atomic Energy Agency (IAEA) within their Incident and Trafficking Database (ITDB) [6]. The system was stablished in 1995 for the “purposes of facilitating information exchange” and to analyse patterns and trends to aid in the identification of potential security threats and vulnerabilities. Such incidents are categorised according to one of three groups: Group 1 (“instances involving a confirmed or likely act of trafficking or malicious use”), Group 2 (“occurrences for which there is insufficient information to determine if it is related to trafficking or malicious use”, i.e., involve stolen or missing material which are indicative of vulnerabilities in security and control systems) and Group 3 (“incidents that are not related to trafficking or malicious use”, i.e., inadvertent and unauthorised possession or shipment of nuclear or radioactive materials). The radionuclides (gamma-ray emitting) most frequently encountered outside of regulatory control are shown in Table 1, alongside their characteristic gamma-ray photopeak energy and application.

A significant degree of annual variation exists across all (three) such categories within the ITDB, with a total of 3686 incidents confirmed by member states between 1993 and 2019, which does not include losses involving the large number of sealed sources used globally for non-destructive testing or within mining and construction applications. Owing to their “detrimental consequences if misused”, the IAEA continues to “underscore the need to improve security measures for such sources” supported by the provision to detect and recover such material. Incidents within “Group 3” are the most common, with an average of 120–160 incidents per year reported by member states over the last decade—of which significant reporting uncertainty/underreporting is invoked to exist [7].

To detect and deter such illicit possession, and therefore protecting populations from the consequences of radioactive material existing outside of regulatory control, a range of detection systems exist across differing scales and for various situational scenarios. The earliest and most recognisable systems for detecting radioactive and nuclear materials are the static arrays of detectors installed at ports, through which vehicles (cars, trucks and containers) successively pass [8,9]. These radiation portal monitor (RPM) systems are classified as “passive”: relying on the detection of a signal emitted from the material of interest (a highly penetrating gamma-ray) without any initial external stimulus to induce a detectable event [10,11]. Such systems are appropriate for the detection of radioactive and nuclear materials emitting radiation in the form of gamma-rays (as shown in Table 1) which represent the greatest hazard from external exposure, however, they are unable to detect radioisotopes that decay through the emission of alpha and/or beta particles. However, it is acknowledged that gamma-emitting material is of the greatest concern as a consequence of its potential use in the production of radiological dispersal devices (RDDs), or “dirty bombs”.

As a consequence of the age of the large number of such systems installed globally, having been developed over a decade ago [9,12], their architecture is based upon low-cost and high efficiency plastic scintillators with large dimensions (for high gamma detection sensitivity), therefore, these systems are “dumb” and cannot afford any spectral information to elucidate as to the nature/composition of the specific radioactive source(s) [13]. To address this limitation and afford an enhanced discrimination between differing radioactive sources and common background materials, work has been undertaken in the USA to develop “advanced spectroscopic portals” (ASPs) based on inorganic or solid-state materials (e.g., NaI, CsI, BGO, CeBr_3_). However, the development and adoption of such systems (namely NaI) for vehicular portal monitoring applications has been limited [14], owing to the size limitations and sensitivity being prohibitive (alongside its greater cost). However, the adoption of these scintillator materials has been observed within pedestrian monitoring infrastructure. Alongside extensive deployments at points of entry across the USA, the UK’s capability “Programme Cyclamen” entered service in 2009 and utilises similar detectors to monitor vehicles at key ports and hub locations around the UK.

These passive systems are in contrast to the less widely utilised “active” detection methods (established for locating fissile material), whereby an incident beam of either low-power neutrons or gamma-rays is directed into a container, vehicle or object of interest. If fissile material (e.g., highly enriched U, or Pu) is present, then a fission event will occur, resulting in the subsequent emission of detectable (secondary) neutrons and high-energy gamma-rays. However, these systems are not extensively deployed, present ethical issues (as the incoming beam could harm people who illegally stowaway inside containers) and are also unable to detect non-fissile (and common gamma-emitting) materials [10,11].

Similar detector setups, comprising large volume and non-spectroscopic systems, are also widespread at key infrastructure sites and transport hubs, such as airports and entertainment venues to screen for humans transporting radioactive material [8], with integrated versions having been deployed at nuclear facilities for decades [15]. As a result of the reduced volume of a person (in comparison to that of a vehicle or shipping container) and the smaller associated detector separations (typically <1 m), the solid-state detectors can be of smaller intrinsic volume while still achieving comparable sensitivities [7]. This high level of sensitivity combined with the non-spectroscopic (“dumb”) nature of the majority of such pedestrian systems consequently results in a significant number of detection events associated with persons having recently undergone medical procedures—about 0.04% of people [12]. However, while the vast majority of the larger vehicular and cargo portal detection capability has yet to fully utilise spectroscopic detectors such as NaI, CsI and CZT because the detector volumes required for personnel type systems are smaller, this infrastructure has seen their initial integration [13]. This static capability is typically supported through the application of small portable detectors, including both spectroscopic and non-spectroscopic personal radiation detectors (PRDs), and larger handheld radioisotope identifiers (RIIDs) capable of analysing a spectrum and fingerprinting the radioactive material [7,13]. Although static pedestrian systems are smaller than vehicular platforms, they are still large and obtrusive pieces of infrastructure [16,17] and do not represent a covert, incorporable, retrofittable or cost-efficient monitoring solution to address the enhanced monitoring requirements of countries and organisations globally. Therefore, this project sought to develop a platform that satisfied these current limitations.

Solid-state (scintillator) detectors, whether plastic, organic or inorganic, represent one means of detecting radiation. While sensitive and (typically) spectroscopic, they are currently of considerable cost (several thousand dollars per detector), which is prohibitive when multiple devices are needed to be incorporated into a single portal and when multiple such systems are required at busy pedestrian locations to stream numerous “lanes” of individuals. To yield such a highly sensitive portal system with a comparable/enhanced spectroscopic capability (fully automated radioisotope identification) and source positional information (during a gamma-emitters transit through the portal), this work developed a multi-detector system comprising an array of (lower-cost yet highly sensitive) gaseous Geiger–Muller (GM) radiation sensors combined with a single solid-state scintillator detector, producing an embedded high-sensitivity yet spectroscopic platform.

## 2. Materials and Methods

### 2.1. Portal System

**Figure 1:**
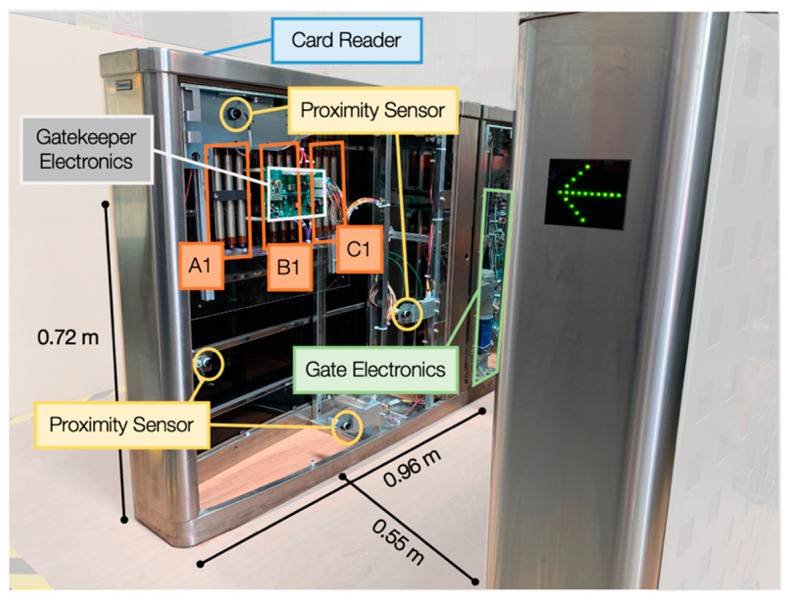
Photograph of the Gunnebo Speed Gate™ entrance control system containing the integrated radiation detection system comprising multiple Geiger–Muller detectors (A1, B1, C1), an array of offset proximity sensors and the single solid-state scintillator (spectroscopy) detector (not shown, on opposing side of Speed Gate™). The corresponding Geiger–Muller detectors (A2, B2, C2) are located directly opposite A1, B1 and C1 within the other upright section of the gate enclosure.

The system selected for embedded detector integration as part of this work was the Gunnebo Ltd. SpeedStile™, part of the Speed Gate™ entrance control portfolio of products. As a product, it is widely deployed globally to provide access control within buildings, airports, transport hubs and at other secure key infrastructure locations. While a number of variants on the product exist, the underlying system components remain largely consistent between models: comprising two parallel (waist-height, 0.95 m tall) units through which a pedestrian user passes as they travel towards the system’s moving gates, which subsequently grant access upon verification from the unit’s integrated card reader (or other identification platform) linked to a central database through a controller area network (CAN) bus. A labelled photograph of the Speed Gate™, with the internal side panels removed, is shown in Figure 1, with a more detailed schematic shown in Figure S1. To prevent against the “tailgating” of one user by another as they transit through the system, the Speed Gate™ is installed with a number of infra-red (IR) proximity (movement) sensors, as shown in Figure 1, to alert the system to likely incursions and consequently deny all entry. As can be observed from the image of the internal workings of Speed Gate™, the majority of the system is comprised of empty space, suitable for detector integration.

For this (standard) Speed Gate™ system, the separation of the two vertical units is 0.55 m, with the distance from the gate entrance (and first IR proximity sensor—the only sensor utilised as part of the Gatekeeper system) to the moving barrier being 0.96 m. A wider barrier system is also manufactured for those using a wheelchair, with an increased separation between the vertical units of 1.2 m. For the development of the detection, localisation and analysis algorithms as part of “Project Gatekeeper”, a typical (reduced) walking speed of 1.0 m/s was used for persons travelling through the system [18].

The accessible and interfaceable electronics, sensors and power distribution present within the Speed Gate™ support the project’s objectives to develop a low-cost sensory platform capable of being retrofitted (covertly) into existing security infrastructure. Furthermore, the widespread adoption of the Gunnebo product range and its deployment at a large number of strategic locations would facilitate its large-scale adoption and implementation. From the gates large printed circuit board (PCB) located within the main “gate electronics”, it is possible to obtain a clean 24-volt DC power supply for the Gatekeeper electronics (and associated radiation sensors), feedback (voltage outputs) from the array of proximity sensors as well as access into the CAN bus to facilitate reporting/alerts through the control/security network administering user access through the Speed Gate™.

### 2.2. Detector Array

**Figure 2:**
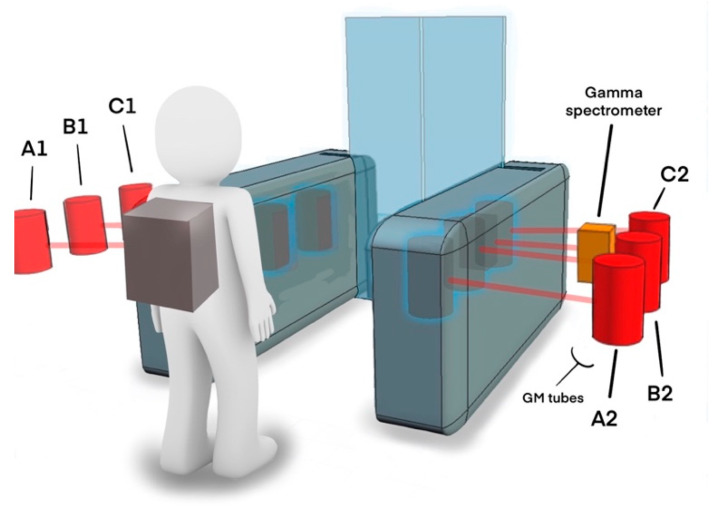
Schematic of the Project Gatekeeper portal system, illustrating the locations of the six distributed Geiger–Muller (GM) detectors (“A1, A2, B1, B2, C1, C2”) and the solid-state miniaturised gamma spectrometer.

As shown in the Figure 1 image of one half of the embedded “Project Gatekeeper” system integrated into the Speed Gate™, three gaseous GM detectors (A1, B1 and C1) are installed at regular separations within the side enclosure and linked to the dedicated Gatekeeper electronics. These three GM detectors are placed opposite three additional detectors (A2, B2, C2) located within the other vertical side of the Speed Gate™ structure (Figure S1), also linked to the dedicated electronics, as shown in Figure 2. The GM sensors serve to provide the initial detection “trigger” to any incident gamma-emitting radiation, from which subsequent localisation and identification can take place.

An inter-detector separation of 20 cm exists between each of the GM modules, of which the T2006/500 pancake type detector from Mirion Technologies (Canberra) Inc. (Canberra UK) was selected. With a high characteristic sensitivity (to α, β and γ radiation) of 3500 counts per minute (58 counts per second—CPS) on exposure to a Cs-137 source with an activity (dose-rate) of 1 mR/hr (~10 µSv/h), an active (window) detection diameter of 44.5 mm and a maximum post-detection deadtime of 50 µs, the module represents the ideal low-cost (<USD 100 per module) sensor for the incorporation of six into each barrier system. The six T2006/500 GM modules each require their own dedicated operational voltage of 500 volts to maintain the sensitive detection plateau which is provided by a dedicated high-voltage (HV) power supply circuit installed within the Gatekeeper PCB, transforming the input 24 V supply sourced from the main gate electronics.

Detection event counting is also undertaken on the Gatekeeper PCB, with the associated voltage spikes on each of the six GM’s HV channels shaped using an integrated pulse modulation circuit before being input into dedicated I/O pins on the PCB’s microcontroller—a MicroPython pyboard v1.1 (George Robotics Ltd.), running the Python (version 3.4) programming language.

Alongside the six gaseous T2006/500 GM modules, the embedded detector array also comprises a single gamma-ray spectrometer, as shown in Figure 2—located inside the gate, close to the position of the movable barriers. The solid-state detector integrated into the Speed Gate™ is the C12137-01 from Hamamatsu Photonics Ltd. The device comprises a 38 × 38 × 25 mm crystal of CsI(Tl) scintillator coupled to a photomultiplier and multi-pixel photon counter (MPPC). Capable of obtaining photon counting information at the desired 10 Hz readout rate (a 50 Hz maximum readout rate is possible with this detection module), the USB-interfaced and powered (150 mA typical current) device is connected to the USB input on the MicroPython pyboard, with control of the detector and its outputs afforded by full Linux device drivers provided by Hamamatsu. While considerably more expensive than the GM units (hence the inclusion of a only single module to reduce system costs), the detector possesses enhanced sensitivity over the GM modules, registering 7000 CPS on exposure to a Cs-137 source with a dose-rate of 10 µSv/h over the energy range of 0–3000 keV (3.0 MeV) [19], thereby suitably encompassing all of the anticipated gamma-ray emission energies shown formerly in Table 1. Although this CsI(Tl) detector does not possess the same spectral resolution as modules made of other materials [20], with a full width at half maximum (FWMH) of 8.5% at 662 keV (Cs-137), it is capable of attaining a low limit of dose-rate detection (0.001 µSv/h) as well as simultaneously converting CPS to dose-rate via its integrated G(E) function.

### 2.3. Radiation Detection and Location

**Figure 3:**
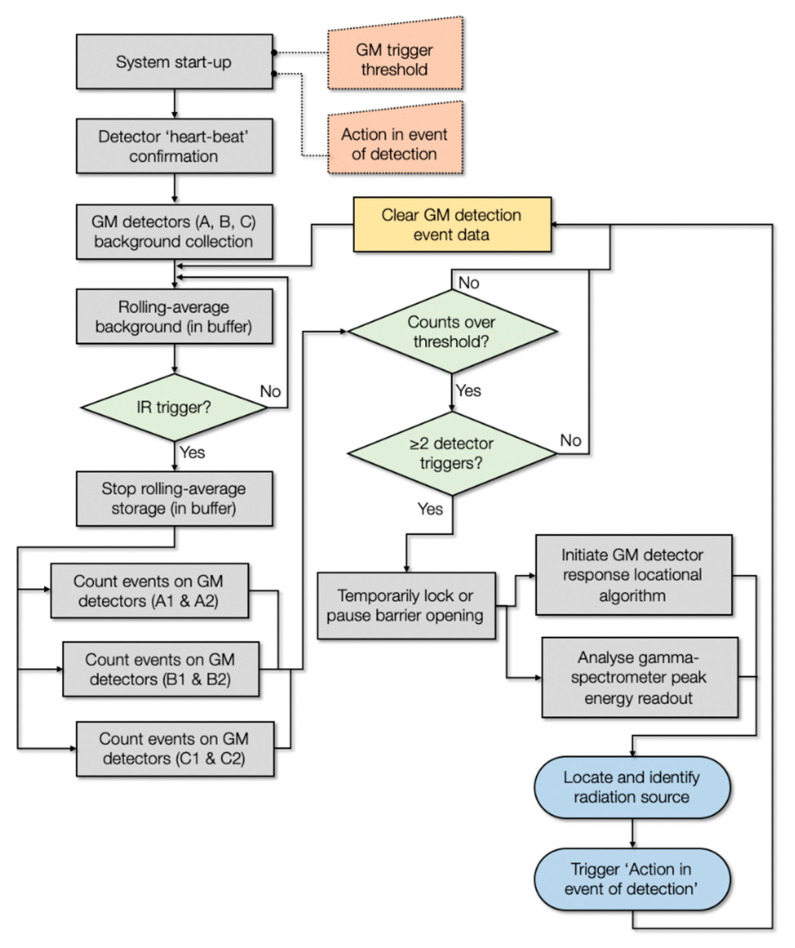
Flowchart of the Project Gatekeeper detection and location algorithm.

The primary detection of radioactive materials entering the sensor-embedded Speed Gate™ is achieved through one (or multiple of) the array of distributed GM detectors. If a detection event is “triggered”, this array of sensors serves also to derive positional information alongside confirmatory and isotopic analysis afforded by the gamma-ray spectrometer. A flowchart of the complete algorithm is shown in Figure 3, comprising the sequence of (i) source incident/occurrence triggering, (ii) source localisation and (iii) source identification.

Upon supply of mains power to the system, the gate and connected Gatekeeper electronics (identified in Figure 1) initialise—with the Speed Gate™ undertaking routine (proximity/IR) sensor and movement checks. To confirm that the GM detectors (×6) and gamma-ray spectrometer are connected and functioning, the Gatekeeper system performs an initial “heart-beat” check by listening for detection events on each module arising from the naturally occurring background. After briefly confirming the presence and correct operation of the devices (with events within predefined tolerances), the system then progresses into the calibration function or returns an error (displayed on its LCD screen) for attention and resolution. During this “start-up” phase, user-defined parameters including (i) action in the event of radionuclide detection (either whether to keep the barrier closed or to allow the user passage with silent alarm) and (ii) sensitivity threshold/source tolerance (e.g., activity above background to trigger system or radionuclides at concentration to not initiate alert, i.e., low concentration of medical isotopes) are captured and implemented by the algorithm from a presets .txt file installed onto the microcontroller. This post-startup calibration/background collection function operates for a period of 180 s, whereby (CPS/10) values for each GM detector are obtained at a frequency of 10 Hz, facilitated by the integrated timing clock on the pyboard module. These CPS/10 measurements from each GM device are sequentially written to the internal memory/buffer of the pyboard, with the system then ready for pedestrian use following this baseline collection period. Resulting from the ability to control the gate through the CAN bus, during this period, the gates remain closed and Speed Gate™ is unable to permit entry.

As shown in the flowchart of Figure 3, the collection of background CPS (as CPS/10) measurements by the six GM counters continues beyond this initial 180-s period to monitor progressive long-term variations in background intensity. The same 10 Hz frequency measurements (CPS/10) are logged onto the buffer, where they are stored for approximately 20 min before being overwritten by new CPS/10 values as part of the “rolling background” function. A single background value for each GM detector is derived, owing to the small sensitivity variations between each device.

**Figure 4:**
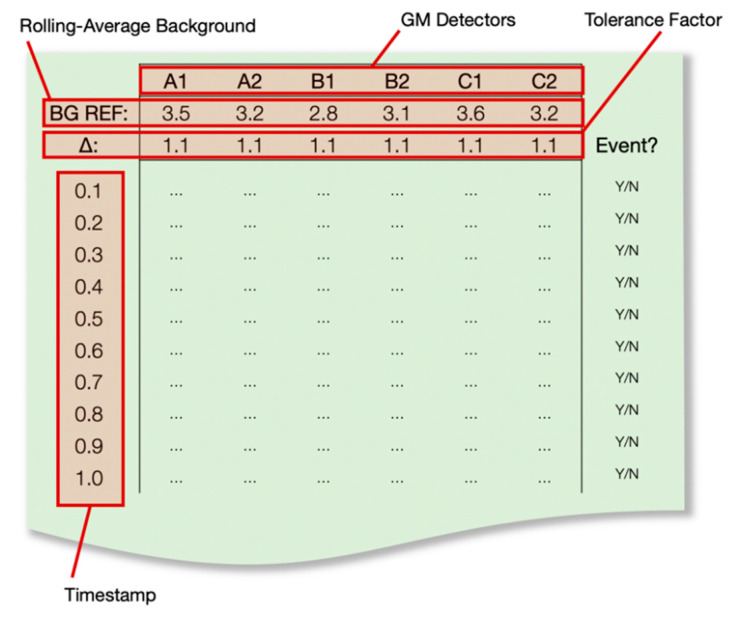
Schematic of the GM detection measurement matrix array populated following the triggering of the IR proximity sensor at the entrance to the Speed Gate™.

The detection algorithm is initiated in each instance by the identification of passing movement by the directly opposing IR proximity sensors at the entrance to the barrier, identified in Figure 1 (and also in the detailed schematic of Figure S1). At this time, the writing of CPS/10 radiation intensity values to the rolling average buffer is paused such that potentially elevated counts do not serve to influence the background rolling average value. Utilising the continually rolling timestamp provided by the pyboard’s in-built clock, the radiation intensity (CPS/10) recorded (at the standard 10 Hz frequency) by each of the six GM detectors is logged to a matrix array alongside (i) the time (in seconds) since the proximity trigger event, (ii) the rolling average (background) value derived for each of the GM modules and (iii) the aforementioned sensitivity threshold/source tolerance, herein termed “tolerance factor” (or Δ)—a multiplication factor applied to each of the background values to define the maximum/trigger value. A schematic of this output matrix is shown in Figure 4. This “tolerance factor” (Δ) can be modified to adjust the triggering sensitivity of the Gatekeeper system, appropriate where (i) considerable environmental variability may exist (a potential consequence of the high intrinsic background from certain construction materials, although this is largely removed through the rolling background functions), and (ii) the frequency of “false positives” may be too great and a facility may therefore wish to suppress this rate, albeit with an increased security risk. However, following testing and the determination of the rate of false positive occurrences, a 10% (Δ = 1.1) “threshold factor” has been shown to represent the ideal value through which to consistently detect even low activity sources while not simultaneously yielding a significant number of detrimental “false positive” events that require human intervention/investigation.

While the intensity values are populated onto the matrix from each GM device, they are compared to each sensor’s rolling average background (termed “BG REF”, as shown in Figure 4) after applying the tolerate (Δ) factor to define the maximum permissible value. The occurrence of a detection “event” above this threshold is identified within the array in the right hand-most column (as “Y” or “N”). Resulting from the ~1 m/s walking pace through the ~1 m length of Speed Gate™, is 1 s of measurements—10 in total (at 10 Hz) are obtained. As shown in the process flowchart of Figure 3, the occurrence of 1 detection event within the array is determined to represent a random event (most likely from cosmic background), with the detection of 2 or more triggers resulting in a confirmed detection event and the further progression of the “Gatekeeper” algorithms. If zero or one trigger event(s) are determined, then a message is sent to the Speed Gate™ electronics (through the CAN bus) to release the barriers and allow entrance, with the array cleared and the system returned to the background collection state.

However, if two or more GM “triggers” are encountered—whether the first two timestamped events (i.e., at 0.1 and 0.2 s) or any two events over the 1 s measuring window—then the two more innovative and advanced aspects of the Gatekeeper system are promptly initiated: (i) locating the source and (ii) spectrally identifying the radioisotope (discussed in Section 2.4). Where two such triggers are encountered, a similar CAN bus message is injected into the Speed Gate™ system to keep the barriers closed indefinitely or delay opening to permit for source characterisation (discussed subsequently in Section 2.4), dependent upon the aforementioned chosen setup parameter.

**Figure 5:**
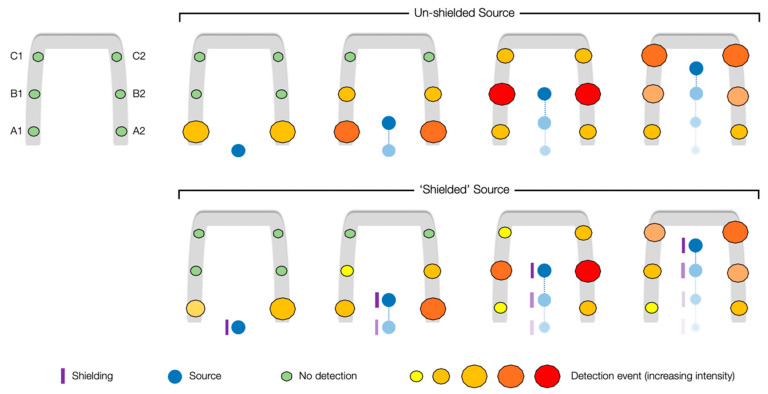
Graphical representation of a radioactive source (unshielded and “shielded”) transiting through the Speed Gate™, inducing elevated count rates on the GM detectors as it moves.

The radioactive source localisation capability is possible as a consequence of the time-integrated variation in detector responses (intensities) to a source at differing (constantly changing) positions within the Speed Gate™, as measured by the array of GM detectors. A graphical representation of this difference in detected radiation intensity by the enclosing sensors of the portal is shown in Figure 5, for both an unshielded and directionally shielded gamma-emitting radioactive source. From earlier work to quantify the influence of biological shielding induced by the human body, it has been established that circa 30% of (Cs-137) gamma radiation is attenuated as a consequence of its interaction with the large volume of strongly attenuating water that comprises the bulk of the human body [21]. Consequently, for a source entering the Gatekeeper-embedded portal concealed on the side of a person, a disparity of up to circa 30% between opposing (A1 and A2, B1 and B2 or C1 and C2) detectors is experienced over a scenario where the radioactive material is unshielded during its path through the Speed Gate™. Hence, through knowledge of the velocity of a person/source progressing through the system (at 1 m/s), the typical detection/trigger response to such a source, and the influence of bodily attenuation yielding an “asymmetrical” detector response, a relative (albeit approximate) source position can be ascribed. As discussed subsequently and corroborated as part of the testing and validation of the Gatekeeper system, such an “emitter localisation” capability is applicable to scenarios where the radioactive source is not only carried within the confines of the entrance control system (i.e., <0.95 m from the ground) on the pedestrian (e.g., within trouser pockets) but also when carried higher from the ground by the pedestrian, above the Speed Gate™ enclosure (e.g., within a jacket pocket, or more likely, inside of a backpack). This is a consequence of the similar gamma radiation “ray” paths and their track to the detectors in the array—whether unimpeded or having sustained some bodily attenuation.

### 2.4. Spectral Identification and Source Attribution

As shown in the sequence flowchart of Figure 3, alongside the initiation of the aforementioned source localisation function and pausing of the barriers release, following the occurrence of two or more GM detection triggers, the simultaneous spectroscopic (isotopic) analysis of the radioactive source contained within the Speed Gate™ is started.

This spectroscopic attribution is performed using the C12137-01 module from Hamamatsu Photonics Ltd., embedded within the barrier and controlled via USB (with associated drivers) by the pyboard. To permit for the rapid identification of the gamma-emitting source, a frequency analysis of the raw channel numbers, output by the solid-state detector over the measurement period is performed on the pyboard as an array, with these being directly calibrated with gamma (photon) energy (in keV). Resulting from the 4096 channels on the device and a detectable energy range of 30–3000 keV (3.0 MeV), a value of 0.73 keV/channel is derived. For radioisotope identification, a commonly adopted “windowing” methodology is hereby adopted [22,23], whereby counts within discrete energy regions of the gamma-ray spectrum (or channels in this instance) are assigned to attribute the presence of a specific radionuclide. The C12137-01 detector channel windows associated with the common gamma-ray emitting radionuclides presented in Table 1, are shown within Table 2. To identify and confirm the presence of an isotope, the number of counts within an isotopes channel window must constitute greater than 10% of the total counts received by the detector, across the entire energy range. In this scenario, a total measurement period for the gamma-ray spectrometer was set at 1 s before an identification of the radioisotope was made.

### 2.5. Result Output

As the Speed Gate™ represents a closed unit, whereby it is not possible to view the LCD screen embedded within the Gatekeeper electronics module, a remote output is therefore necessary to view the serial (printed) outputs of the algorithm on the pyboard and the associated status of the gate. To provide this status visualisation, a serial window is used on a remote computer, connected to the pyboard through a USB cable. While this represents a suitable interim mechanism through which to observe the systems status on a small/local scale, full integration of the Gatekeeper module into the SpeedGate™ will utilise communications via the CAN bus to the central control network, and/or via a Bluetooth-compatible application (“App”) to nearby personnel.

### 2.6. System Validation

To validate the performance of the full Project Gatekeeper infrastructure, including both the detection system and aforementioned algorithms, it was tested with a suite of radioactive sources of differing isotopic composition and intensity as well as being carried on through the Speed Gate™ portal on different parts of the body. A summary of the validation parameters is given in Table 3 and is represented schematically within Figure S2.

As shown in Table 3 and also pictorially in Figure S2, to test the capability, response and sensitivity of the Gatekeeper system to radioactive material carried at the different (bodily) positions likely to be encountered, an extensive number of simulated transits were undertaken. All of the source (“isotope”) compositions and dose rates were carried in each “source position”—of which a sub-division is made based upon its height (above the ground)/position relative to the top of the barrier enclosure (0.95 m). Those “source height” conditions of 0.70–0.90 m are analogous to a concealment of material within pockets, while those at 0.96–1.20 m are conversely representative of a radioactive source carried, for example, in a coat pocket (Front, Side: Left and Side: Right) or within a pedestrians backpack (Rear). In each scenario, once within the Speed Gate™, the separation of the radioactive source to any one of the GM detectors is never greater than 0.35 m, and a maximum of 0.70 m from the furthest module, all of which are mounted within the Speed Gate™ at a height of 0.85 m from the ground. It is of note that this small (<1 m) source–detector separation is in contrast to the known half-distance of the characteristic Cs-137 gamma-ray of approximately 70 m [24].

## 3. Results and Discussion

### 3.1. Radioactive Source Initial Detection and Localisation

**Figure 6:**
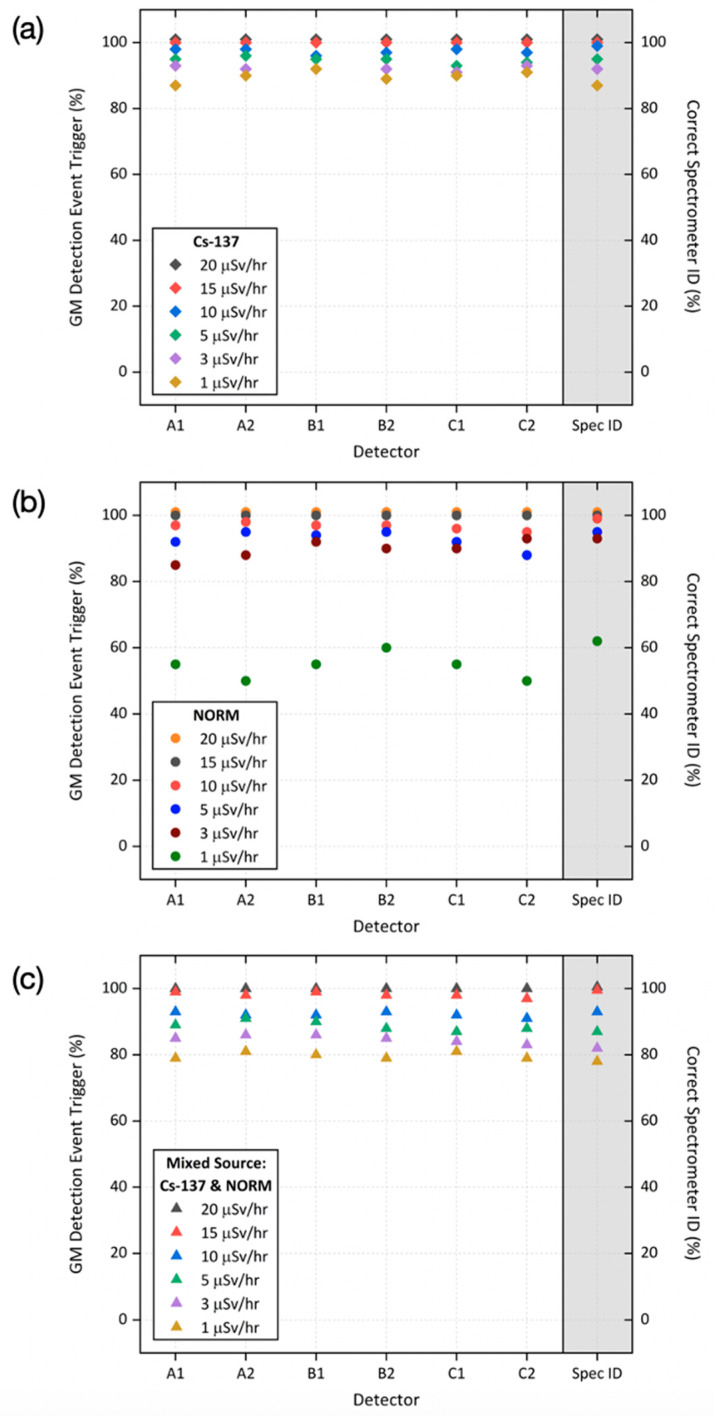
Sensitivity analysis of the integrated platform, detailing its ability to detect and spectrally identify sources of differing dose-rate (activity) levels, compositions and “on-person” positions, as detailed in Table 3, for sources of (**a**) Cs-137, (**b**) NORM and (**c**) mixed Cs-137 and NORM.

Through applying the aforementioned standard 0.1 (10%) “tolerance factor” above the rolling average background as the detection threshold for each GM device to trigger and initiate the subsequent system response, a sensitivity analysis of the GM detectors within the system to initiate these triggers is presented in Figure 6, for (a) Cs-137, (b) NORM sources and (c) mixed Cs-137 and NORM, each of differing intensities. Alongside the percentage incidence of the GM detectors (A1, A2, B1, B2, C1, C2) delivering a trigger when sources of distinctive composition and varying strength are walked through the Speed Gate™, Figure 6 (both plots) also shows the incidence of the gamma-ray spectrometer outputting the correct isotope identification following counting.

As shown in Figure 6a, for the Cs-137 sources, both the highest dose-rate (activity) material can be detected by all six GM devices 100% of the time and similarly spectrally identified in all instances. For all other Cs-137 sources (1, 3, 5 and 10 μSv/h), they could be detected by all six GM detectors in greater than 85% of the occurrences. However, no situation existed where the presence of the source was not sensed by at least two of the GM detectors to result in a positive trigger and progression of the algorithm. The gamma-ray spectrometer and associated channel analysis algorithm was capable of “fingerprinting” the radionuclide within the currently permissible 1 s analysis time in greater than 84% of instances (better for higher-activity sources). A greater counting time would, however, yield better counting statistics (increased signal to noise) from which to base the source identification.

The Geiger–Muller sensitivity analysis from the equivalent NORM source testing of the detection capabilities using the same intensity sources is presented in Figure 6b. From this plot, it is similarly shown that for the greatest dose-rate sources (10, 15 and 20 μSv/h) that a >90% detection rate is associated with all six of the gaseous detectors—with this rate, >90% and >82% for the 5 μSv/h and 3 μSv/h sources, respectively. However, a marked reduction in “all detector” triggering is connected to the 1 μSv/h source—with all detectors confirming a trigger event on 49% of instances. As in the Cs-137 detection scenario, there were no transits of the NORM source through the Speed Gate™ detector array that did not result in less than two trigger events. A similar high accuracy in radioisotope identification was also attained for the higher dose-rate sources of the NORM material: correct in >95% of instances for 3, 5, 10, 15 and 20 μSv/h trials.

However, only a 62% occurrence of correct spectral identification was associated with the 1 μSv/h NORM source, markedly lower than that of Cs-137. Such a reduced identification rate via the pyboard’s channel analysis of the gamma-ray spectrometers output can likely be invoked as a result of the energy of the gamma-ray emissions coupled with the reduced detector photon conversion efficiency at such higher energies (Bi-214 at 1760 keV and Tl-208 at 2610 keV), in comparison to the lower energy of Cs-137 (663 keV), where greater efficiencies exist.

The results of the mixed (Cs-137 and NORM) source Gatekeeper testing are similarly presented in Figure 6c. Such results are analogous to the Cs-137 sensitivity analysis shown in Figure 6a, being characterised by the similarly high GM detection event trigger percentage, whereby for all such mixed sources (1, 3, 5, 10 and 20 μSv/h), they were detected by all six GM detectors in greater than 79% of the occurrences. Of note, there existed no situation whereby the presence of the source was not sensed by at least two of the GM detectors to consequently yield a positive trigger and progression of the remainder of the Gatekeeper algorithm, as detailed in Figure 3. The gamma-ray spectrometer and associated channel analysis algorithm was capable of “fingerprinting” the mixed nature of the material within the currently permissible 1 s analysis time in greater than 78% of instances (improved for higher-activity sources). As for both the Cs-137 and NORM sources, a greater counting time would yield better counting statistics (increased signal to noise) from which to base such a source identification.

While it would be beneficial to increase the integration time (from 1 s) of the gamma-ray spectrometer to enhance the radioisotope fingerprinting and the correct spectrometer identification, in order to not impede/detriment the operation of the Speed Gate™ system, this current iteration of the Gatekeeper software does require any additional time to undertake the spectral measurements other than the 1 s over which the user transits through the portal. This preservation of high levels of pedestrian flow through the Speed Gate™ was a foundational requirement of this project.

**Figure 7:**
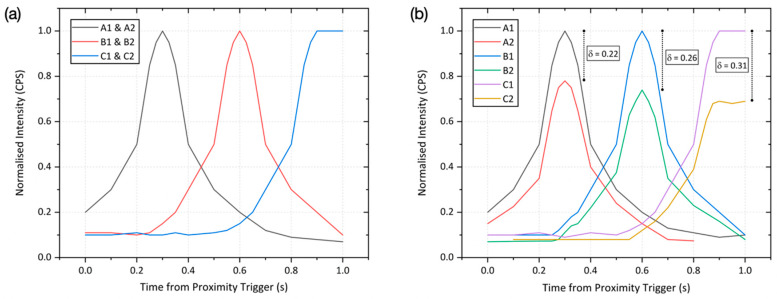
Geiger–Muller (GM) detector responses to a 5 μSv/h Cs-137 source transiting through the Gatekeeper system: (**a**) “unshielded” and (**b**) “shielded” through being located on the left hand side of the user.

While at least two of the GM detectors triggered during all test scenarios due to above threshold counts (therefore initialising the spectroscopic identification function), resulting from the failure of not all six detectors to register elevated counts for low-intensity sources, the source localisation function could not, therefore, be undertaken. The response of each of the six Geiger–Muller detectors to a 5 μSv/h Cs-137 source transiting through the system under different conditions is further examined and quantified in Figure 7. Within Figure 7a, the Cs-137 source is held on a vertical mount so to not introduce any shielding influences, and progressively moved along the central axis of the gate towards the barriers at the 1 m/s walking pace. As expected, the same response (intensity and profile) was experienced by each GM unit that comprises the three opposing detector pairs. From the plot, three distinct peak events are apparent following the triggering of the IR proximity sensor (time = 0) and commencement of measurements, as the source moves closer to the GM detector before soon moving away again. The levelling of the final peak in Figure 7a is the result of the source being held adjacent to this detector pair for an extended period as a consequence of the Speed Gate’s™ barriers being held closed following positive source detection (and subsequent isotopic identification). It is this temporal intensity variation that facilitates the source localisation.

In contrast to the strongly symmetric peaks evidenced by the progression of an “unshielded” source through the detector array, as shown in Figure 7a, the comparable movement (at 1 m/s) of a “shielded” source carried on the left hand side of a person through the gate produces significantly different results, as shown in Figure 7b. Unlike the former plot, where identical radiation intensities were obtained by both detectors in each pair, differences (δ) between the opposing detectors varied between 22% and 31%, a consequence of the significant shielding contribution introduced by the person and which aligns with the previously invoked reduction owing to bodily attenuation [21]. By advancing the “unshielded” source scenario, it is such a time-resolved disparity between the opposing detectors that permits for real-time radioactive source localisation during its transit through the Speed Gate™, embedded with the Gatekeeper system.

### 3.2. Radioactive Source Identification

**Figure 8:**
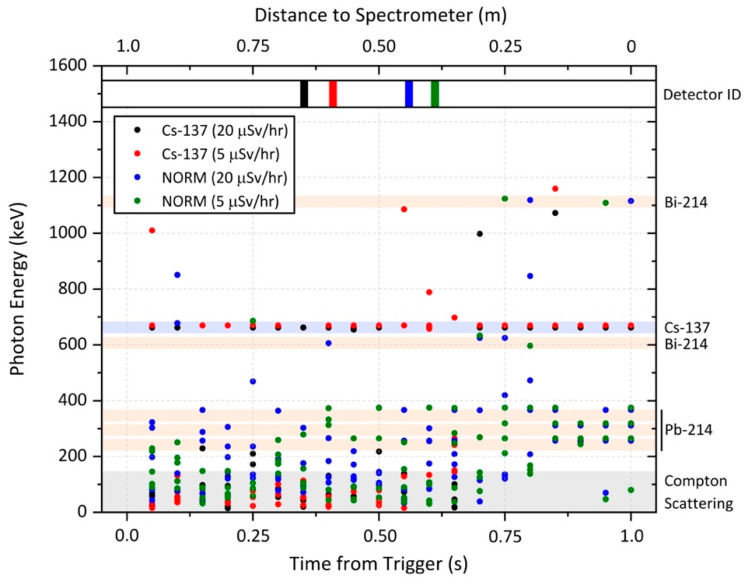
Analysis of the integrated spectrometer response to four trial radioactive sources (Cs-137 and NORM) transited through the gate. The “time from trigger” represents the duration from the IR sensor at the front.

Following triggering two or more of the array of six GM detectors installed within the Speed Gate™ (with intensity above the predetermined threshold), identification of the radioactive source contained within the gate is subsequently performed using the USB readout from the solid-state Hamamatsu C12137-01 scintillator detector, as shown in the schematic of Figure 3. A plot of the photon counting and channel window identification performed by the embedded C12137-01 module is presented in Figure 8, for two composition sources, each of differing intensity (dose-rate).

Over the standard 1 s counting period from the “time from trigger” (with GM triggering of the spectral identification algorithm occurring as the source enters the Speed Gate™ and with the source travelling through the Speed Gate μSv/h at the typical 1 m/s), Figure 8 details the individual photon responses (herein converted to photon energy, in keV) with time and transit. As can be seen for the two Cs-137 sources (5 μSv/h and 20 μSv/h) in Figure 8, detection events occur within the designated channels immediately after the detector is activated, with event (channel) frequency further increasing, alongside the signal to spectral noise improving over the duration of the measurement period. This high count rate enables a rapid “detector ID” of the radionuclide (defined as 10% of the total counts within the designated window), shown by the bars at the top of the plot, with the 20 μSv/h Cs-137 source identified within 0.35 s and the 5 μSv/h Cs-137 source spectrally classified within 0.40 s of the detector initialising. A similar, albeit marginally smaller, increase in the windowed count rate/event frequency is observed for both the 5 μSv/h and 20 μSv/h NORM sources, as shown in Figure 8. While the highlighted windows do not show a high incidence of counts following the gamma-ray spectrometers initiation (at “time from trigger” = 0), the number of detection events within these windows rapidly increases until a radioisotope identification is possible. The higher dose-rate 20 μSv/h NORM source was successfully identified in an average time of 0.56 s, with the 5 μSv/h NORM source recognised in 0.6 s from the spectrometer measurement commencement. This slight delay in provenancing the NORM sources in comparison with the Cs-137 is a result of the single monoenergetic emission from Cs in contrast to the multiple photopeaks arising from the NORM material—many of which occur on the shoulder of the Compton edge or at high energies where the inherent detector efficiency is reduced.

However, it should be noted that in these four test scenarios, it was not required to undertake measurements for the entire 1 s duration in order to obtain source identification, therefore demonstrating the efficiency of the setup and algorithm(s).

### 3.3. “False Positive” Alarm Activiation

A system such as this rapidly loses its efficacy if a large number of “false positive” events are recorded by the detection platform, which subsequently require manual/additional intervention/screening activities on persons to be undertaken. To ascertain the frequency of such incorrect detection events, a number of random “source-free” or “blank” transits through the Speed Gate™ were undertaken during the aforementioned Radioactive Source Initial Detection and Localisation (3.1) and Radioactive Source Identification (3.2) experiments. Of the >400 source-less passes through the Speed Gate™, only two “false positive” events occurred. Such a low incident rate (0.5%) is therefore indicative of a sensitive, yet highly specific, detection system that would not demand sustained human intervention to disregard these detection events.

## 4. Conclusions and Future Work

“Project Gatekeeper” has sought to produce a low-cost, covert and retrofittable detection system capable of being integrated into an existing Gunnebo Speed Gate™ portal infrastructure to rapidly and accurately detect gamma-emitting radioactive material, alongside providing locational and full spectroscopic identification on the source. The system was shown to be capable of detecting low-activity radioactive sources of differing compositions, down to 1 μSv/h for sources composed of NORM or Cs-137. By utilizing a number of more cost-effective GM detectors complimented by a single scintillator detector, the “Project Gatekeeper” architecture represents a readily deployable and highly covert system with the ability to be fully integrated into existing entrance control portals located at airports, train stations, public venues or high security locations.

A summary of the current status of various aspects of the “Project Gatekeeper” system is presented in Table 4, alongside the potential future system properties that it is hoped to achieve.

Future work will look at improving the detection efficiency and sensitivity of the system. This will be achieved through the integration of additional and/or more sensitive Geiger–Muller detectors into the system and refining the GM-based locational algorithms. Complimentary improvements to the gamma-ray spectrometer aspects of the Gatekeeper system will work to enhance the speed to (correct) source identification, alongside incorporating faster readouts and most-sensitive spectroscopic modules. Through innovations in low-cost and high-sensitivity scintillator detector materials/devices, an analogous array of such solid-state spectroscopic devices could soon replace the GM units, therefore, further enhancing the capabilities of the embedded Gatekeeper platform.

## Figures and Tables

**Table 1:** Common gamma-ray emitting radionuclides that are most frequently encountered outside of regulatory control, their gamma-ray emission energies and application(s), from [7].

Isotope	Gamma Energy (keV)	Application
Co-60	1173 & 1332	Nuclear
Tc-99m	140	Medical
I-131	365	Medical
Cs-134	604 & 796	Nuclear
Cs-137	663	Nuclear/Medical
Ir-192	317 & 468	Industrial
Am-241	59	Industrial
U-235	186	Nuclear
NORM *	609 & 1760 (Bi-214), 2610 (Tl-208) and 241, 295 & 351 (Pb-214)	Natural

* NORM: naturally occurring radioactive material—containing U (and Th), of which their daughter products are the primary gamma-emitting radionuclides.

**Table 2:** Derived detection (channel) windows for the commonly occurring radionuclides listed in Table 1, for the Hamamatsu C12137-01 gamma-ray spectrometer.

Isotope	Gamma Energy (keV)	Channel Window(s)
Co-60	1173 & 1332	850–860 & 968–978
Tc-99m	140	97–107
I-131	365	262–272
Cs-134	604 & 796	435–445 & 576–586
Cs-137	663	478–488
Ir-192	317 & 468	226–236 & 336–346
Am-241	59	38–48
U-235	186	130–140
NORM	609 & 1760 (Bi-214), 2610 (Tl-208) and 241, 295 & 351 (Pb-214)	440–450 & 1279–1289 (Bi-214), 1900–1910 (Tl-208) and 170–180, 210–220 & 251–261 (Pb-214)

**Table 3:** Summary of the Project Gatekeeper system validation parameters. (N.B. the isotopes of each dose-rate (activity) were used in each of the four stated source positions and heights).

Isotope	Dose Rate (µSv/h)	Source Position	Source Height (m)
Cs-137	1 3 5 10 15 20	Side: Left Side: Right Front Rear	0.70–0.90 (within gate) 0.96–1.20 (above gate)
NORM	1 3 5 10 15 20	Side: Left Side: Right Front Rear	0.70–0.90 (within gate) 0.96–1.20 (above gate)
Mixed (Cs-137 and NORM)	1 3 5 10 15 20	Side: Left Side: Right Front Rear	0.70–0.90 (within gate) 0.96–1.20 (above gate)

**Table 4:** Summary of the current properties of the Gatekeeper platform alongside the intended future performance characteristics.

System Property	Current Value/Detection Limit	Future Value/Detection Limit
Detection Sensitivity: Cs-137 *	1 μSv/h	<0.5 μSv/h
Detection Sensitivity: NORM *	1 μSv/h	<0.5 μSv/h
Detection Sensitivity: Mixed Source *	1 μSv/h	<0.5 μSv/h
Rate of “False Positive” Occurrence	0.66%	<0.1%
Radiation Detection Type	Gamma-rays (γ)	Not required
Detectable Gamma Energy Range (MeV)	0.03–3.00	Not required

* Defined as the ability to detect the presence of a source within the Speed Gate™ by the Gatekeeper system on at least 99% of instances.

## Data Availability

Data arising from the validation aspects of this work are available to download from the repository located at: http://dx.doi.org/10.17632/zz8zzrdsj7.1.

## Supplementary Materials

The following are available online at https://www.mdpi.com/1424-8220/20/10/2957/s1, Figure S1: Schematic of the integrated Gate Keeper detection system. Figure S2: Visualisation of the Gate Keeper system validation methodology.
