# Supplementary material for: Project Gatekeeper: An Entrance Control System Embedded Radiation Detection Capability for Security Applications
Source: Sensors (Basel). 2020 May 23;20(10):2957. doi: 10.3390/s20102957 (PMC7285256; doi:10.3390/s20102957)
Supplement: Supplementary file 1 [file sensors-20-02957-s001.pdf]

Supplementary Material for:

Project Gatekeeper: An entrance control system embedded radiation detection capability for security applications.

Peter G. Martin [1*], Yannick Verbelen [1], Elia Sciama Bandel [2], Mark Andrews [3], Thomas B. Scott [1]

[1] Interface Analysis Centre, School of Physics, HH Wills Physics Laboratory, University of Bristol, Tyndall Avenue, Bristol BS8 1TL, UK.
[2] School of Physics, HH Wills Physics Laboratory, University of Bristol, Tyndall Avenue, Bristol BS8 1TL, UK
[3] Gunnebo UK Ltd, Fairfax House, Pendeford Business Park, Wolverhampton WV9 5HA, UK.
* Correspondence: peter.martin@bristol.ac.uk; Tel.: +44-(0)-117-42-82541

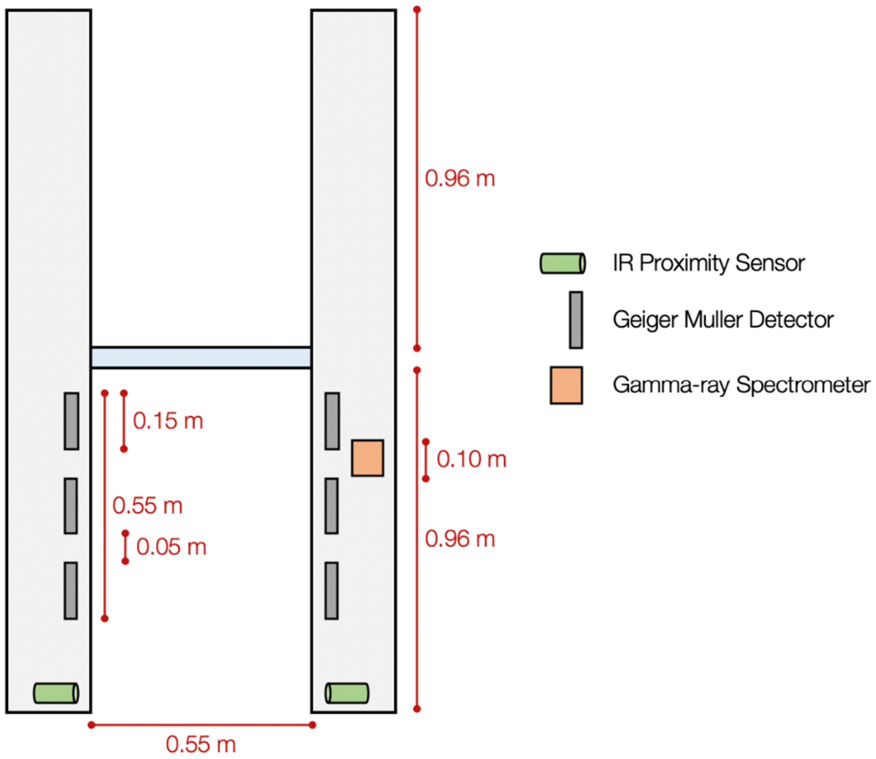

Figure S1. Schematic of the integrated Gate Keeper detection system, embedded within the Speed Gate™ entrance control system.

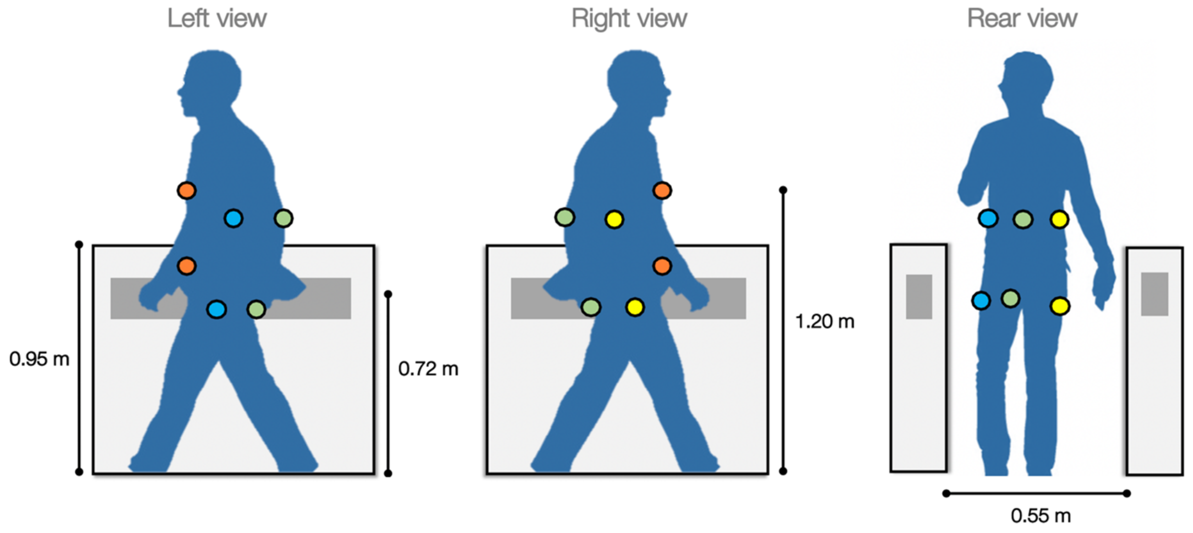

Figure S2. Schematic of the Gate Keeper system validation methodology; utilising sources of differing isotopic compositions and activities (as shown in Table 3), located on different parts of the body as it transited through the detection system. The detector arrays (Geiger-Muller tubes on both sides, plus the gamma-ray spectrometer on the right-hand side only) are contained within the side panels of the Speed Gate™ within a region centred 0.72 m from the ground – as highlighted above. A number of the sources were located on the body above the walls of the barrier system, with other located within its confines – closer to the detector array. The location convention of the sources is thus; Orange = front, Blue = left, Yellow = right, Green = rear.
